# Effects of noradrenaline and phenylephrine on cerebral oxygen saturation during cardiopulmonary bypass in cardiac surgery

**DOI:** 10.1113/EP092387

**Published:** 2025-01-23

**Authors:** Edouard Marques, Etienne J. Couture, Jean S. Bussières, Stephan Langevin, Paul Poirier, Pierre Voisine, Manon Caouette, Patrice Brassard

**Affiliations:** ^1^ Institut Universitaire de Cardiologie et de Pneumologie de Québec–Université Laval Québec Canada; ^2^ Department of Kinesiology, Faculty of Medicine Université Laval Québec Canada

**Keywords:** cardiac surgery, cerebral oximetry, noradrenaline, phenylephrine, vasopressor

## Abstract

Cardiopulmonary bypass (CPB) in cardiac surgery is associated with a high risk of postoperative neurological complications. Perioperative use of vasopressors is common to counteract arterial hypotension in this setting. However, use of α‐agonist vasopressors has been associated with cerebral desaturations. Given that reductions in cerebral oxygen saturation ($S_{cO_{2}}$) can increase postoperative neurological dysfunction, we aimed to investigate the impact of noradrenaline (NA) and phenylephrine (PE) on $S_{cO_{2}}$ during the CPB period of a cardiac surgery in 36 patients scheduled for an elective cardiac surgery. Patients were randomized to the intra‐operative use of either NA or PE. During CPB, mean arterial pressure (MAP) was elevated pharmacologically to predefined thresholds of 60 and 80 mmHg, while CPB flow was kept constant. The $S_{cO_{2}}$ values were recorded for 5 min per MAP threshold. The MAP increased adequately between thresholds of 60 and 80 mmHg (NA, 59 ± 3 vs. 81 ± 3 mmHg and PE, 61 ± 4 vs. 81 ± 3 mmHg; *P* ˂ 0.01). The $S_{cO_{2}}$ decreased between pressure thresholds of 60 and 80 mmHg (NA, 70 ± 11 vs. 69 ± 11 mmHg and PE, 64 ± 11 vs. 63 ± 11 mmHg; *P* ˂ 0.01). Reduction in $S_{cO_{2}}$ did not differ between vasopressors. The mean relative decrease in $S_{cO_{2}}$ across groups was 2.0% (95% confidence interval: 0.6 to 2.1). Elevation in MAP mediated solely by vasopressors induces significant decreases in $S_{cO_{2}}$ during cardiac surgery under CPB. However, their impact on $S_{cO_{2}}$ remains clinically non‐significant according to current guidelines.

## Abstract (highlights)

- **What is the central question of this study?**
  What is the impact of an elevation in mean arterial pressure, mediated by noradrenaline or phenylephrine, on cerebral oxygenation in patients undergoing cardiac surgery with cardiopulmonary bypass.
- **What is the main finding and its importance?**
  The findings indicate that relative cerebral oxygenation decreased by 2% between 60 and 80 mmHg with noradrenaline and phenylephrine. These statistically significant decreases in cerebral oxygenation have no clinical impact. These results support the safety of increasing mean arterial pressure to the upper limit of recommended mean arterial pressure under cardiopulmonary bypass.

## 1 INTRODUCTION

The use of cardiopulmonary bypass (CPB) during cardiac surgery is associated with a high risk of neurological complications. For instance, postoperative stroke has been diagnosed in 1%–8% of patients (Lo et al., 2022) and postoperative cognitive dysfunctions have been reported in ≤40% of patients after cardiac surgery with CPB (Greaves et al., 2020). Although part of the risk is inherent to the procedure, cardiac surgery patients are now older and present risk factors for neurological complications (Hogue et al., 1999; Mullan et al., 2020). Given that most of these patients undergoing general anaesthesia also have structural or functional cardiac deficits, vasopressors and inotropes are often used to maintain adequate systemic blood pressure and cerebral blood flow (CBF) (Guinot et al., 2023). Studies have reported associations between reductions in cerebral saturation of oxygen ($S_{cO_{2}}$) monitored using near‐infrared spectroscopy and concomitant use of α‐agonist vasopressors, such as phenylephrine (PE) and noradrenaline (NA), with variations in $S_{cO_{2}}$ averaging −0.8% to −14.0% (Bombardieri et al., 2021; Brassard et al., 2014, 2009; Froese et al., 2020; Holmgaard et al., 2019; Larson et al., 2020; Meng et al., 2012; Meng, Sun, Zhao et al., 2024; Moerman, Denys et al., 2013; Nissen et al., 2010). PE acts as a pure α_1_‐agonist, whereas NA acts primarily as an α_1_‐agonist with some activity on α_2_‐ and β_1_‐adrenergic receptors (Overgaard & Dzavik, 2008).

Cerebral desaturations are typically greater in non‐cardiac surgery than in cardiac surgery with CPB (Meng, Sun, Zhao et al., 2024). This phenomenon could be associated with a decrease in cardiac output mediated by vasopressor use. For instance, experiments at steady state have shown that each 1% decrease in cardiac output induces a reduction of 0.35% in CBF, which impacts $S_{cO_{2}}$ values (Meng et al., 2015). However, the mechanism underlying cerebral desaturations mediated by vasopressors in a state of fixed cardiac output under CPB has yet to be documented clearly. Animal models have shown increases in the superior cervical ganglion activity following provoked systemic hypertension but not hypotension (Cassaglia et al., 2008). Such activation in cerebral sympathetic activity might induce cerebral vasoconstriction to prevent hyperperfusion, potentially leading to decreases in CBF and $S_{cO_{2}}$. Theories involving a direct vasoconstriction mediated by α‐receptors in the brain vasculature have also been proposed (Froese et al., 2020). A potential deleterious effect of α‐agonist vasopressors on $S_{cO_{2}}$ is worrisome, because new evidence shows associations between cerebral desaturations and increases in postoperative cognitive dysfunction and delirium following cardiac surgery (Tian et al., 2022).

Mean arterial pressure (MAP) recommendations during CPB are to aim between 50 and 80 mmHg (Wahba et al., 2020). Targeting 80 mmHg instead of 50 mmHg in all patients might involve substantially higher vasopressor doses, with uncertain clinical benefits. Therefore, considering the increase in patient comorbidities, the high risk of neurological complications arising from the surgical intervention and the association between $S_{cO_{2}}$ and postoperative neurological dysfunctions, it appears reasonable to limit any clinical intervention that might negatively impact $S_{cO_{2}}$ during cardiac surgery.

As of today, very few research groups have aimed to differentiate the impact of different vasopressors on $S_{cO_{2}}$. The objective of this prospective randomized study was to determine the impact of an elevation in MAP, mediated by NA or PE, on $S_{cO_{2}}$ in patients undergoing cardiac surgery with CPB. We hypothesized that both vasopressors would induce a reduction in $S_{cO_{2}}$_,_ which would be greater in the NA group. Indeed, a previous study comparing NA and PE showed that NA induced significant reductions in frontal lobe $S_{cO_{2}}$ of patients with diabetes when compared with patients without diabetes during CPB. Reductions of $S_{cO_{2}}$ were not significant in the PE group between these two groups (Brassard et al., 2014).

## 2 MATERIALS AND METHODS

### 2.1 Ethical approval

This study was conducted at the Institut universitaire de cardiologie et de pneumologie de Québec–Université Laval (IUCPQ‐UL), an academic affiliated and tertiary referral centre in cardiac surgery, between 1 February 2021 and 30 March 2022. Privacy rights of human subjects have been observed, and all patients provided informed written consent before participating in this study. The study was approved by the Research and Ethic Board (2014‐2290) according to the principles established by the *Declaration of Helsinki* (except for registration in a database).

### 2.2 Eligibility criteria

This single‐centre study was conducted in a clinical setting context of patients scheduled for elective cardiac surgery under CPB at our institution. After consent, 36 patients were randomized to the use of either NA (*n* = 18) or PE (*n* = 18), in a ratio of 1:1, as the main vasopressor during surgery. A randomized sealed envelope was opened after consent to ensure preparation of the allocated vasopressor before surgery. Implementation of the randomization protocol was supervised by one of the co‐investigators (E.M.). Patients ranging from 35 to 80 years old, both males and females, undergoing elective cardiac surgery for primary aortic or mitral valve replacement in addition to coronary artery bypass graft were included. The need for a vasopressor therapy for arterial hypotension (MAP ˂ 60 mmHg) following initiation of CPB was necessary to begin the study protocol.

To prevent confounding factors affecting $S_{cO_{2}}$, the following exclusion criteria were implemented upon evaluation of patients before consent: (1) presence of documented cerebrovascular disease (history of transient ischaemic attack, stroke or carotid stenosis); (2) left ventricular ejection fraction ˂35%; (3) known grade IV or V aortic atherosclerosis; (4) diagnosis of type 1 or 2 diabetes; (5) diagnosis of myocardial infarction in the prior 30 days; and (6) non‐elective surgery (Cohen, 2008).

To maintain the highest level of stability in variables influencing $S_{cO_{2}}$ during data collection, the following interventions led to post‐consent exclusion: (1) administration of preoperative oxygen before baseline measurements; (2) diagnosis of grade IV or V aortic atherosclerosis on the perioperative transoesophageal echocardiography; (3) use of del Nido cardioplegia; and (4) administration of blood transfusions in the priming of CPB or after initiation of the CPB.

### 2.3 Study design and data collection

After consent and randomization, medical records of the patients were screened preoperatively to collect demographics and characteristics of the patients in each group. Age, sex, body mass index, relevant comorbidities, cardiovascular medications, cardiac function parameters, laboratory tests and types of surgery were noted.

Next, patients were taken to the operating room for induction of anaesthesia. Given the need for a normal arterial oxygen saturation ($S_{cO_{2}}$), no oxygen supplementation was allowed before the first pre‐induction measurement to obtain the baseline arterial partial pressure of oxygen ($P_{aO_{2}}$) and $S_{cO_{2}}$. The following monitoring was installed for all patients on arrival in the operating room: a five‐lead ECG monitor, pulse oximetry (Nellcor SpO_2_ Module, Medtronic, Brampton, ON, Canada) and a cerebral oximetry monitor (INVOS^TM^ 5100C, Medtronic, Brampton, ON, Canada). Then, an invasive arterial pressure monitoring system was installed via the radial artery for continuous systemic arterial pressure monitoring and arterial blood gas sampling. After installation of the radial artery catheter, the following measurements were taken simultaneously to establish individual baseline profiles prior to induction: MAP via the radial artery, left‐ and right‐sided $S_{cO_{2}}$ via the cerebral oximetry monitor, $S_{aO_{2}}$ via the pulse oximeter, and $P_{aO_{2}}$, arterial partial pressure of carbon dioxide ($P_{\mathrm{aC}O_{2}}$) and haemoglobin (Hb) measured via sampling of arterial blood gases.

After preoxygenation, general anaesthesia was induced with propofol (1.0 mg/kg) and sufentanil (1–3 µg/kg). Patients were intubated after administration of rocuronium (1.0–1.5 mg/kg) and complete muscular relaxation. Anaesthesia was maintained with sevoflurane without continuous infusion of opioids throughout the procedure. Controlled ventilation was established for all patients, with an end‐tidal CO_2_ between 35 and 42 Torr. After induction, a central venous catheter and a pulmonary artery catheter were installed in the right internal jugular vein. In the eventuality of arterial hypotension (MAP ˂ 60 mmHg), the allocated vasopressor, either NA or PE, was administered. Upon initiation of CPB, the following parameters were targeted: a fixed pump flow of 2.2–2.4 L/min/m^2^, a temperature of 34°C–35°C, normocapnic $P_{\mathrm{aC}O_{2}}$ values between 35 and 42 Torr, and $P_{aO_{2}}$ values between 115 and 130 Torr. Arterial blood gases were monitored continuously with a blood parameter monitoring system (CDI 550, Terumo Cardiovascular Systems, Ann Arbor, MI, USA). Anaesthesia during CPB was maintained with 2% sevoflurane administered through the CPB oxygenator for all patients and propofol (40–50 µg/kg/min) at the discretion of the anaesthesiologist. In the eventuality of arterial hypotension (MAP ˂ 60 mmHg), the allocated vasopressor therapy was either initiated after induction of anaesthesia and pursued during CPB or initiated at the beginning of CPB.

**FIGURE 1:**
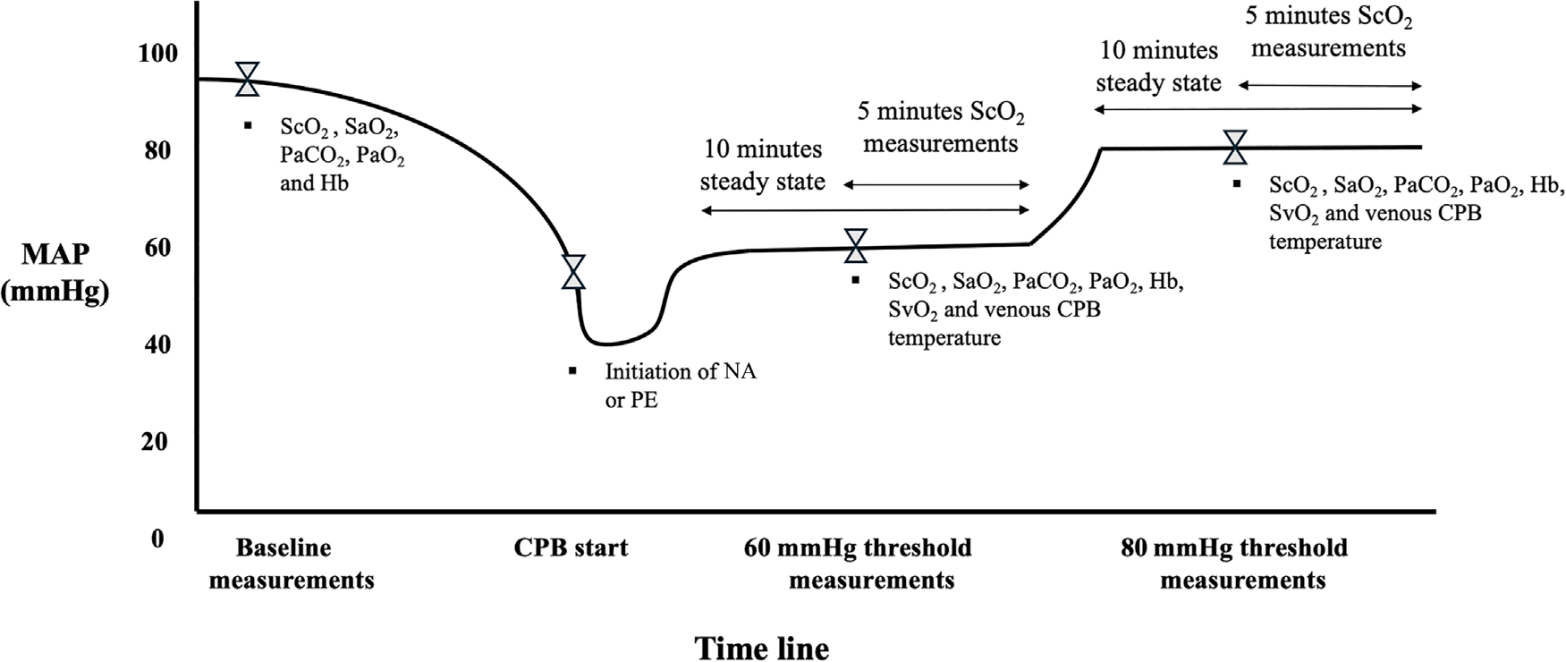
Time line of measurements as a function of MAP thresholds. Baseline measurements of $S_{cO_{2}}$, $S_{aO_{2}}$, $P_{\mathrm{aC}O_{2}}$, $P_{aO_{2}}$ and Hb were performed before induction of anaesthesia, with no oxygen supplementation. The allocated vasopressor was initiated if MAP was <60 mmHg at induction of anaesthesia or upon initiation of CPB. After initiation of CPB, the following parameters were targeted: a fixed pump flow of 2.2–2.4 L/min/m^2^, a temperature of 34°C–35°C, normocapnic $P_{\mathrm{aC}O_{2}}$ values between 35 and 42 Torr, and $P_{aO_{2}}$ values between 115 and 130 Torr. Using the allocated vasopressor, a first MAP threshold of 60 mmHg was obtained for 10 min. After the first 5 min of steady state, the following data were collected once: $S_{cO_{2}}$, $S_{aO_{2}}$, $P_{\mathrm{aC}O_{2}}$, $P_{aO_{2}}$, Hb, $S_{vO_{2}}$ and venous CPB temperature. Subsequently, $S_{cO_{2}}$ values were recorded every minute for the last 5 min of steady state. The same protocol was performed for measurements at the 80 mmHg threshold. Abbreviations: CPB, cardiopulmonary bypass; Hb, haemoglobin; MAP, mean arterial pressure; NA, noradrenaline; $P_{\mathrm{aC}O_{2}}$, arterial partial pressure of carbon dioxide; $P_{aO_{2}}$, arterial partial pressure of oxygen; PE, phenylephrine; $S_{aO_{2}}$, systemic arterial oxygen saturation; $S_{cO_{2}}$, cerebral saturation of oxygen; $S_{vO_{2}}$, central venous oxygen saturation.

Data collection during CPB involved MAP‐based thresholds of 60 and 80 mmHg for each patient. After initiation of CPB, if MAP was <60 mmHg, the allocated vasopressor was pursued or initiated using a perfusion titrated by the perfusionist to reach a target MAP of 60 mmHg at first. A MAP steady state for 5 min was obtained at targeted CPB pump flow/$P_{aO_{2}}$/$P_{\mathrm{aC}O_{2}}$ and temperature parameters before beginning data collection. After 5 min at MAP steady state, the following variables were recorded: MAP via the radial artery catheter, $P_{aO_{2}}$, $P_{\mathrm{aC}O_{2}}$ and Hb via arterial blood gases, venous temperature of the CPB circuit, and venous saturation ($S_{vO_{2}}$) via venous blood gas analysis from the CDI device of the CPB. Moreover, left‐ and right‐sided $S_{cO_{2}}$ were collected every minute for the next 5 min, while maintaining all other parameters at steady state. Therefore, total steady‐state duration was 10 min (Figure 1).

The same protocol and measurements were realized after elevation of MAP to 80 mmHg using the same vasopressor. Of note, MAP was manipulated only through the vasopressor infusion and not the CPB pump flow. These MAP targets were chosen because they resembled the range used in previous studies (Hagen et al., 2016; Vedel et al., 2018). They are also commonly used at our institution and remain inside the thresholds recommended by the guidelines (Wahba et al., 2020).

### 2.4 Cerebral oximetry monitor

Cerebral oximetry allows for continuous non‐invasive measurements of frontal lobe $S_{cO_{2}}$. Measurements are possible owing to the different spectra of absorption of deoxygenated and oxygenated Hb in the near‐infrared wavelengths by using the principles of the Beer–Lambert law, similar to pulse oximetry (Ghosh et al., 2012). For this study, the INVOS cerebral oximetry system (INVOS^TM^ 5100C, Medtronic, Brampton, ON, Canada) was used in all patients. The device consists of two rectangular captors displaying light‐emitting optodes that are applied 3 cm over the eyebrows, allowing bilateral frontal lobe $S_{cO_{2}}$ measurements. The INVOS monitor uses continuous‐wave interrogation and assumes a fixed cerebral arteriovenous blood ratio of 25:75. Wavelengths of 730 and 810 nm are emitted from the two light‐emitting optodes, spaced by 30 and 40 mm from their light detector (Moerman, Vandenplas et al., 2013). Such placement of optodes allows for the subtraction of light reflection from tissues superficial to the brain. Data are reported from the oximetry monitor as an index of the ratio of oxyhaemoglobin to total haemoglobin in the cerebral capillaries to estimate regional cerebral capillary O_2_ saturation (Brassard et al., 2009, 2010).

### 2.5 Statistical analyses

The primary objective was to determine the impact of NA and PE on $S_{cO_{2}}$ assessed with cerebral oximetry at MAP targets of 60 and 80 mmHg during the CPB period during a cardiac surgery. Based on preliminary and previously published findings, a total of 16 participants per group were necessary to detect a reduction of 9% ± 10% in $S_{cO_{2}}$ between the NA and PE groups, to be considered statistically significant with a power of 0.80 and a *P*‐value < 0.05 (Brassard et al., 2014). Taking into consideration a possibility of dropouts (exclusion of data for analysis owing to clinical actions necessary for patient safety, such as manipulation of the CPB pump flow, $P_{\mathrm{aC}O_{2}}$, body temperature or depth of anaesthesia during infusion of vasopressors precluding steady‐state levels of MAP during infusion of vasopressors), inclusion of a total of 18 participants per group was decided. Of note, post‐consent exclusions were not replaced by recruiting new patients. Demographic data were presented for continuous variables as the mean ± SD or as the median with associated interquartile range according to the variable distributions. Categorical variables were presented as absolute or relative frequencies. Mean $S_{cO_{2}}$ ($S_{cO_{2}\mathrm{mean}}$) was calculated from right and left $S_{cO_{2}}$ values. Baseline characteristics were analysed with Student's *t*‐tests for normally distributed variables and Mann–Whitney *U*‐tests for the remaining variables. Fisher's exact test was used for categorical variables. Two‐way ANOVAs were used to evaluate the interaction between MAP targets and vasopressor groups. Analyses were conducted using GraphPad Prism v.10.0.0 for Windows. Values of *P* < 0.05 were considered statistically significant. To increase the generalizability and robustness of our results, a bootstrapping approach with 1000 resamples was used specifically for $S_{cO_{2}}$ values to create a larger distribution for both groups and time points prior to analysis. A two‐factorial ANOVA was computed to examine the effects of group, time and their interaction on the bootstrapped mean estimates. This analysis was conducted using RStudio (v.4.3.0).

## 3 RESULTS

### 3.1 Patient exclusions

Among the 36 patients who gave consent, eight were excluded following initiation of the research protocol (Figure S1). In the NA group, five patients were excluded because of grade IV atherosclerosis of the aorta (*n* = 1), use of del Nido cardioplegia (*n* = 1), administration of blood transfusion at CPB start (*n* = 1) and arterial hypertension precluding use of vasopressor therapy (*n* = 2). In the PE group, three patients were excluded because of preoperative administration of oxygen (*n* = 1), administration of blood transfusion at CPB start (*n* = 1) and arterial hypertension precluding the use of vasopressors (*n* = 1). The study protocol was completed for all the other 28 patients.

### 3.2 Patient demographics

The mean age was comparable between groups (Table 1). Both groups were composed mostly of male patients. Comorbidities were similar between groups, apart from arterial hypertension, which was more frequent in the PE group. Medications for the treatment of cardiovascular disease, preoperative laboratory tests and echocardiographic measures of left ventricular ejection fraction did not differ between groups. The European System for Cardiac Operative Risk Evaluation (EuroSCORE) II was comparable in both the NA and PE groups. Among surgery types, more patients underwent single valve replacement in the NA group.

**TABLE 1:** Demographics and baseline preoperative values of study groups.

Characteristics	Noradrenaline (*n* = 13)	Phenylephrine (*n* = 15)	*P*‐value
Age (years)	66.8 ± 10.0	68.3 ± 7.0	0.61
Male (%)	11 (84.6)	14 (93.3)	0.46
BMI (kg/m^2^)	26 ± 3	28 ± 4	0.08
Comorbidities			
Active smoker Arterial hypertension Dyslipidaemia Atrial fibrillation Previous myocardial infarction	0 (0.0) 7 (53.8) 7 (53.8) 2 (15.3) 2 (15.3)	1 (6.7) 14 (93.3) 13 (86.7) 3 (20.0) 4 (26.7)	1.00 0.03 0.09 1.00 0.7
Medication			
ACEI/ARB β‐Blockers Aspirin Clopidogrel DOAC	6 (42.9) 6 (42.9) 7 (53.8) 0 (0.0) 2 (15.3)	11 (73.3) 7 (46.7) 12 (80.0) 0 (0.0) 4 (26.7)	0.4 1 0.2 1 0.65
EuroSCORE II (%)	1.5 ± 1.5	1.8 ± 1.6	0.34
LVEF (%)	56 ± 7	59 ± 9	0.23
Laboratories			
HbA_1C_ (%) Troponin I (ng/L) Total cholesterol (mmol/L) Creatinine (mmol/L)	5.4 ± 0.3 10 [8, 13] 4.3 ± 1.5 78 ± 15	5.5 ± 0.4 10 [7, 28] 3.6 ± 0.8 86 ± 14	0.52 0.61 0.09 0.14
Surgery type			
CABG Single valve Combined	6 (42.9) 5 (38.4) 2 (15.3)	9 (60) 0 (0.0) 6 (40.0)	0.71 0.01 0.22
Preoperative values			
MAP (mmHg)	97 ± 12	111 ± 8	˂0.01
$S_{cO_{2}}$ L (%) $S_{cO_{2}}$ R (%) $S_{cO_{2}\mathrm{mean}}$ (%)	74 ± 9 73 ± 8 73 ± 8	66 ± 12 66 ± 12 66 ± 11	0.06 0.08 0.07
$S_{aO_{2}}$ (%)	98 ± 2	98 ± 1	0.61
$P_{aO_{2}}$ (Torr)	88 ± 15	95 ± 17	0.22
$P_{\mathrm{aC}O_{2}}$ (Torr)	34 ± 4	35 ± 5	0.36
Hb (g/L)	132 ± 41	136 ± 15	0.74

*Note*: Values are presented as the median [interquartile range], mean ± SD or *n* (%).

Abbreviations: ACEI, angiotensin‐converting enzyme inhibitor; ARB, angiotensin receptor blocker; BMI, body mass index; CABG, coronary artery bypass graft; DOAC, direct oral anti‐coagulant; EuroSCORE II, European System for Cardiac Operative Risk Evaluation II; Hb, haemoglobin; HbA_1C_, glycated haemoglobin; LVEF, left ventricular ejection fraction; MAP, mean systemic arterial pressure; $P_{\mathrm{aC}O_{2}}$, arterial partial pressure of carbon dioxide; $P_{aO_{2}}$, arterial partial pressure of oxygen; $S_{aO_{2}}$, systemic arterial oxygen saturation; $S_{cO_{2}}$ L, left cerebral oxygen saturation; $S_{cO_{2}\mathrm{mean}}$, mean cerebral oxygen saturation; $S_{cO_{2}}$ R, right cerebral oxygen saturation.

### 3.3 Patient baseline characteristics

In accordance with a higher prevalence of arterial hypertension in the PE group, MAP was also higher at baseline measurements (*P* ˂ 0.01). The $S_{cO_{2}\mathrm{mean}}$ values were not different in the NA and PE groups. Other parameters measured at baseline, such as $S_{aO_{2}}$, $P_{aO_{2}}$, $P_{\mathrm{aC}O_{2}}$ and Hb, did not differ between cohorts (Table 2).

**TABLE 2:** Study variables as a function of the allocated vasopressor and mean arterial pressure thresholds.

Variable	Noradrenaline (*n* = 13)		Phenylephrine (*n* = 15)		PSEM	*P*‐value		
	60 mmHg	80 mmHg	60 mmHg	80 mmHg		Drug	Time	D × T^a^
MAP (mmHg)	59 ± 3	81 ± 3	61 ± 4	81 ± 3	2.63	0.19	˂0.01	0.15
$S_{cO_{2}\mathrm{mean}}$ (%)^b^	*70 ± 11*	*69 ± 11*	*64 ± 11*	*63 ± 11*	*1.32*	*˂0.01*	*˂0.01*	*0.23*
$P_{aO_{2}}$ (Torr)	131 ± 17	127 ± 15	128 ± 19	123 ± 16	11.50	0.54	0.15	0.98
$P_{\mathrm{aC}O_{2}}$ (Torr)	41 ± 1	42 ± 2	40 ± 2	41 ± 2	1.48	0.07	0.01	0.48
Hb (g/L)	114 ± 10	116 ± 11	107 ± 14	108 ± 14	1.55	0.14	˂0.01	0.01
CPB flow (L/min/m^2^)	2.3 ± 0.1	2.3 ± 0.1	2.3 ± 0.1	2.3 ± 0.1	0.03	0.21	0.26	0.27
Temperature (°C)	34.3 ± 0.3	34.5 ± 0.5	34.6 ± 0.4	34.0 ± 0.3	1.34	0.31	0.72	0.57
$S_{vO_{2}}$ (%)	80 ± 5	81 ± 4	78 ± 5	77 ± 7	3.12	0.13	0.85	0.27

*Note*: Values are presented as the mean ± SD.

Abbreviations: CPB, cardiopulmonary bypass; Hb, haemoglobin; MAP, mean systemic arterial pressure; $P_{\mathrm{aC}O_{2}}$, arterial partial pressure of carbon dioxide; $P_{aO_{2}}$, arterial partial pressure of oxygen; PSEM, pooled standard error of the mean; $S_{cO_{2}\mathrm{mean}}$, mean cerebral oxygen saturation; $S_{vO_{2}}$, central venous oxygen saturation.

^a^ D × T = Drug × Time interaction effect.

^b^ Values in italics represent bootstrapped mean estimates.

### 3.4 Mean arterial pressure thresholds and cerebral saturation of oxygen

**FIGURE 2:**
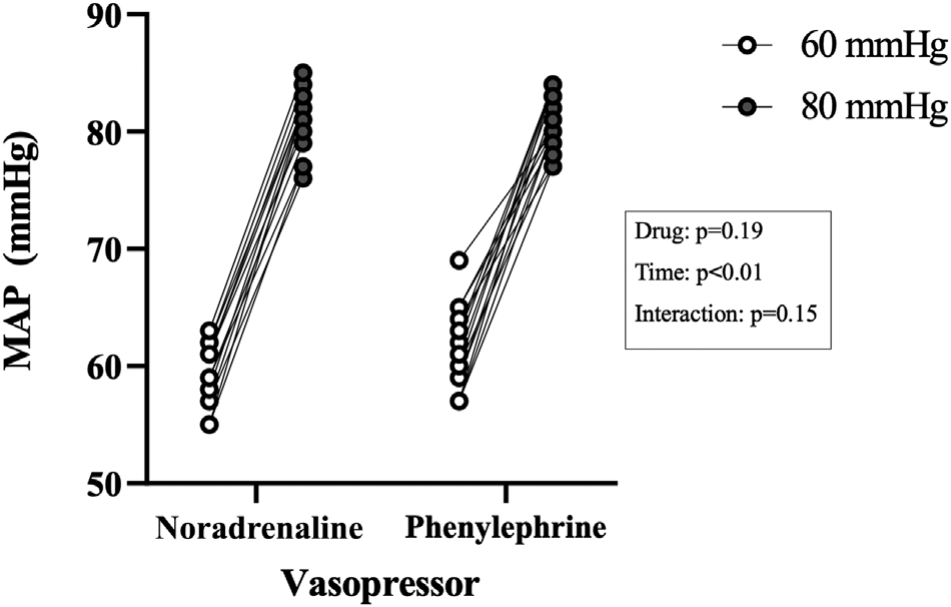
The MAP as a function of randomized vasopressor and MAP thresholds. On the left, MAP values after noradrenaline perfusion for 60 and 80 mmHg MAP targets. On the right, MAP values after phenylephrine perfusion for 60 and 80 mmHg MAP targets. The MAP was comparable across groups for the same time measurements (*P* = 0.19) but was significantly different when compared between the 60 and 80 mmHg thresholds (*P* ˂ 0.01). Abbreviation: MAP, mean arterial pressure.

**FIGURE 3:**
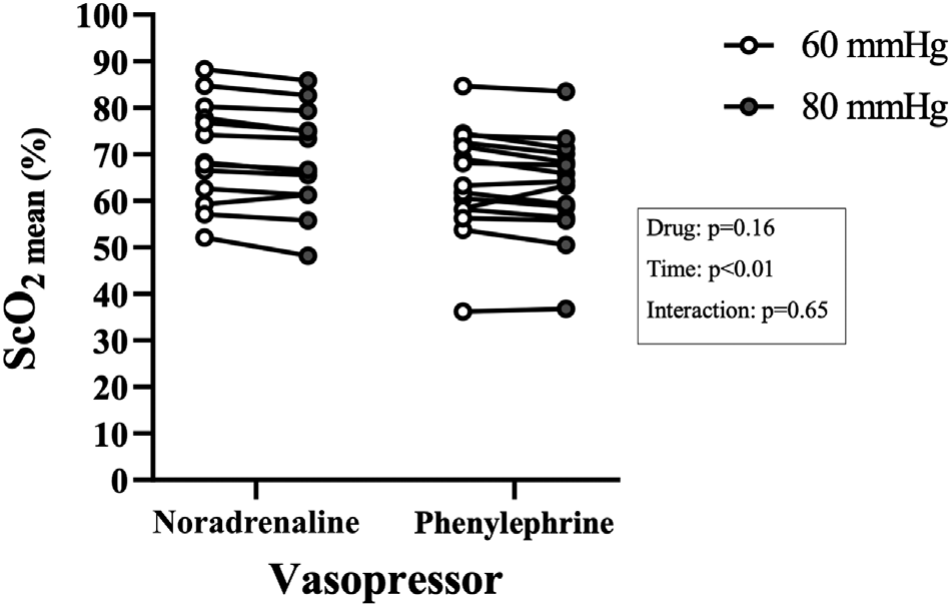
The $S_{cO_{2}\mathrm{mean}}$ as a function of randomized vasopressor and MAP. On the left, $S_{cO_{2}\mathrm{mean}}$ after noradrenaline perfusion for 60 and 80 mmHg MAP targets. On the right, $S_{cO_{2}\mathrm{mean}}$ after phenylephrine perfusion for 60 and 80 mmHg MAP targets. The $S_{cO_{2}\mathrm{mean}}$ values were comparable across groups for the same time measurements (*P* = 0.16) but were significantly different when compared between the 60 and 80 mmHg thresholds (*P* ˂ 0.01). Abbreviations: MAP, mean arterial pressure; $S_{cO_{2}\mathrm{mean}}$, mean cerebral oxygen saturation.

According to the study design, ANOVA measurements showed significant differences in MAP according to the 60 and 80 mmHg pressure thresholds [*F*(1, 26) = 843.5, *P* ˂ 0.01]. The MAP values were comparable across groups for the same pressure–time measurements [*F*(1, 26) = 1.79, *P* = 0.19; Figure 2; Table 2]. Vasopressor doses needed to achieve targeted MAP steady states were also different between 60 and 80 mmHg (NA, 0.12 ± 0.13 vs. 0.25 ± 0.23 µg/kg/min, *P* ˂ 0.01; and PE, 0.37 ± 0.45 vs. 0.99 ± 1.1 µg/kg/min, *P* = 0.02). Two‐factorial ANOVA on the bootstrapped mean estimates showed significant differences in mean $S_{cO_{2}}$ values between groups [*F*(1, 3996) = 245.1, *P* ˂ 0.01]. Analyses also showed significant reductions in $S_{cO_{2}}$ between thresholds of 60 and 80 mmHg [*F*(1, 3996) = 4539.2, *P* ˂ 0.01; Figure 3]. Reductions in $S_{cO_{2}}$ between 60 and 80 mmHg were comparable for both NA and PE, because the interaction between vasopressor groups and pressure–time $S_{cO_{2}}$ measurements was non‐significant [*F*(1, 3996) = 1.5, *P* = 0.23]. The mean relative decrease in $S_{cO_{2}}$ across groups was 2.0% (95% confidence interval 0.6 to 2.1).

### 3.5 Cardiopulmonary bypass parameters at steady state

Among the parameters kept at steady state, $P_{aO_{2}}$, venous temperature and flow of CPB were comparable in both groups and at both pressure–time measurements. The $P_{\mathrm{aC}O_{2}}$ was similar between groups but showed a significant increase between MAP thresholds of 1.1 Torr [95% confidence interval −1.9 to −0.2, *F*(1, 26) = 7.04, *P* = 0.01]. Haemoglobin also exhibited an increase of 1.7 g/L between MAP thresholds [95% confidence interval −2.5 to −0.8, *F*(1, 26) = 15.9, *P* ˂ 0.01], but did not differ between the NA and PE groups.

## 4 DISCUSSION

The aim of the present study was to determine the impact of NA and PE on $S_{cO_{2}}$ assessed by cerebral oximetry at MAP targets of 60 and 80 mmHg during the CPB period of a cardiac surgery. Patients acted as their own controls for $S_{cO_{2}}$ variations, allowing for individualized interpretation of variables. For both vasopressors, relative mean $S_{cO_{2}}$ values decreased by 2.0% between measurements at 60 and 80 mmHg. The reduction in $S_{cO_{2}}$ aligned with significant increases in vasopressor perfusion doses. Thus, $S_{cO_{2}}$ decreased significantly when MAP was elevated solely by means of vasopressor therapy, and the vasopressor impact on $S_{cO_{2}}$ was comparable for NA and PE. Our results align with the previously stated hypothesis that both vasopressors would induce a reduction in $S_{cO_{2}}$; however, reductions in $S_{cO_{2}}$ were not greater in the NA group as expected.

This challenges the results of a previously published study where NA seemed to have a greater impact on $S_{cO_{2}}$ than PE when used under CPB in patients with diabetes (Brassard et al., 2014). Although the population in the present study did not include patients with diabetes, our results have failed to demonstrate that either NA or PE induced a greater cerebral desaturation load when used to raise MAP under CPB. This might be attributed to a population effect, because patients with diabetes have been shown to exhibit enhanced responsiveness to NA and might depict altered cerebrovascular function (Ciavarella et al., 1994). Experimental studies have shown that when compared with controls during periods of exercise, patients with diabetes present with reduced CBF and cerebral oxygenation despite similar uptake of lactate and glucose (Kim et al., 2015). Limited ability to increase cardiac output and impaired cerebral vasodilatation have been hypothesized as contributory mechanisms to these findings (Kim et al., 2020). However, there is also a possibility that both NA and PE exert a similar impact on $S_{cO_{2}}$ during CPB and that the previous study by Brassard et al. (2014) did not have enough power to detect these results (NA diabetics *n* = 6, non‐diabetics *n* = 8 vs. PE diabetics *n* = 8, non‐diabetics *n* = 9). Although not on cerebral haemodynamics, multiple studies have shown that NA and PE present similar α‐agonist‐mediated effects on systemic haemodynamics in the obstetrics and intensive care populations (Morelli et al., 2008; Ngan Kee et al., 2015), and to our knowledge, no studies highlight a greater potential for venoconstriction with NA or PE. The main difference between those two vasopressors rests in the presence of a β‐agonist effect of NA, which mainly affects cardiac output by increasing contractility and heart rate (Ducrocq et al., 2012). Nonetheless, the β‐agonist effect of NA yields no impact on cardiac output under CPB, because systemic blood flow is regulated by the CPB pump and not the heart.

### 4.1 Impact of vasopressors on $S_{cO_{2}}$ in the literature

Recent literature has shown valuable results about the impact of α‐agonist vasopressors on $S_{cO_{2}}$ in multiple categories of patients. Typically, studies in awake patients depicted higher reductions in $S_{cO_{2}}$ with α‐agonist vasopressors than studies in anaesthetized patients (Meng, Sun, Zhao et al., 2024). Healthy volunteers who were administered PE exhibited a paradoxical reduction of 11% in $S_{cO_{2}}$ when MAP was elevated by 22% across grouped studies. Potential explanations for this phenomenon include a decrease in cardiac output caused by both bradycardia and ventriculo‐arterial uncoupling, as cardiac afterload increases without changes in contractility. In addition to the decrease in cardiac output, a compensatory mechanism of cerebral autoregulation or sympathetic activation might induce cerebral vasoconstriction in response to the elevation in MAP. The same phenomenon was found in patients under general anaesthesia for non‐cardiac surgeries when MAP was manipulated pharmacologically with PE, but of lesser magnitude, with reductions in $S_{cO_{2}}$ averaging 3.4%. This discrepancy might be explained by the attenuation of cerebral autoregulation mediated by volatile anaesthetics (Meng, Sun, Rasmussen et al., 2025).

In a CPB context, studies investigating the impact of NA and PE showed even smaller reductions in $S_{cO_{2}}$ (−0.2% to −1.8%) following vasopressor administration (Hagen et al., 2016; Moerman, Denys et al., 2013; Sperna Weiland et al., 2017). We believe that reductions in $S_{cO_{2}}$ under CPB might be smaller owing to exclusion of cardiac output as a potential variable, which is a strength of the present study. Therefore, mechanisms behind these results can be associated more precisely with a cerebral vasoconstriction mediated by cerebral autoregulation, a direct sympathetic effect of either NA or PE on the cerebral vasculature or a limitation from our cerebral oximetry monitoring to interpret contamination from scalp and cranial signals.

### 4.2 Hypothesis for underlying mechanisms

The rationale for the effect of vasopressors on $S_{cO_{2}}$ is still debated. In humans, it is commonly agreed on that NA and PE do not cross an intact blood–brain barrier (BBB) (Greenfield & Tindall, 1968). However, studies in humans have shown disruption of the BBB during CPB, and the intensity of BBB disruption at imagery has been linked to postoperative neurocognitive dysfunction. (Abrahamov et al., 2017). Direct passage of vasopressors through a damaged BBB could induce deleterious effects, and NA has been found at increased levels in the cerebrospinal fluid of traumatic brain injury patients receiving exogenous NA (Mautes et al., 2001). No human studies have reported adverse neurological effects of such passage, although an increase in cerebral metabolism, CBF and vasogenic oedema could, theoretically, be possible (MacKenzie et al., 1976; Moller et al., 2004). More research is warranted on that matter, and to our knowledge, no literature exists on the effects of PE through a damaged BBB.

Moreover, animal studies were able to demonstrate direct elevation in cerebral sympathetic nervous activity in response to transient increases in MAP induced by NA (Froese et al., 2020). Although a direct impact of vasopressors on the cerebral vasculature has yet to be reported in human studies, sympathetic innervation is found in both human and animal cerebral vasculature (Brassard et al., 2017; Koep et al., 2022). The cerebral sympathetic innervation could be a complementary mechanism to cerebral autoregulation and might contribute to protective cerebral vasoconstriction in response to systemic hypertension, thus preventing hyperperfusion (Brassard et al., 2017). However, the conditions for activation of this cerebral sympathetic innervation remain unclear. A recent study showed that in healthy participants, an exercise‐induced elevation in MAP did not have an impact on brain NA spillover (a neurochemical method to estimate total and regional sympathetic nervous activity). It was concluded that transient increases in MAP during acute exercise do not engage cerebral sympathetic activity in these healthy participants (Tymko et al., 2024). Next, others have implied limitations of the cerebral oximetry monitoring system in its ability to interpret data containing information from the extracerebral layers (Eleveld et al., 2023; Meng, Sun, Zhao et al., 2024; Strangman et al., 2014). In fact, experiments have shown that scalp ischaemia induced decreases in $S_{cO_{2}}$ values, which could happen with high infusion doses of vasopressors and might have influenced the values obtained in our study (Davie & Grocott, 2012). Measurements by the INVOS cerebral oximetry monitor also assume a fixed arteriovenous blood ratio in the brain of 25:75. It is not excluded that vasopressor‐induced cerebral desaturations reflect a change in the contribution of arterial versus venous blood measured by the monitor (Ogoh et al., 2011). Finally, a decrease in $S_{cO_{2}}$ caused by cerebral autoregulation‐mediated vasoconstriction in response to increases in MAP is also possible. Moerman et al. (2015) have described paradoxical decreases in $S_{cO_{2}}$ following administration of PE during CPB, which were present only in patients with functional cerebral autoregulation. This could be a potential explanation, because cerebral autoregulation seems to be preserved in most patients during CPB (Vranken et al., 2017).

### 4.3 Impact of comorbidities and CPB variables

The recruited patients presented a relatively low burden of comorbidities in the context of cardiac surgery. Cohorts were similar apart from arterial hypertension, which was more prevalent in the PE group. Notably, a tight control was maintained over parameters deemed to have a significant impact on $S_{cO_{2}}$. Of importance, sevoflurane, which was used to maintain anaesthesia in all patients, does not induce dysfunctions in cerebral autoregulation at the doses used in this study (Juhasz et al., 2019). To eliminate the confounding factor of cardiac output on CBF and $S_{cO_{2}}$, the present study was conducted in the context of CPB. Analyses showed that among groups and MAP thresholds the CPB flow did not change, ensuring a stable surrogate of cardiac output. Temperature and $P_{aO_{2}}$, which can induce changes in $S_{cO_{2}}$ parameters, remained stable throughout the protocol, minimizing their impact as confounding factors on $S_{cO_{2}}$ as well (Johnston et al., 2003; Nevin et al., 1987). The $P_{\mathrm{aC}O_{2}}$ showed a mean increase of 1.1 Torr between thresholds, which was similar in NA and PE groups. $P_{\mathrm{aC}O_{2}}$ is a well‐known mediator in the cerebral vasculature and has been shown to induce direct changes in CBF. Hypercapnia is known to increase CBF by 6%–8% per Torr $P_{\mathrm{aC}O_{2}}$, whereas hypocapnia decreases CBF by 3%–4% per Torr $P_{\mathrm{aC}O_{2}}$ (Hoiland et al., 2019). Rasmussen et al. (2007) showed that controlled increases in $P_{\mathrm{aC}O_{2}}$ were associated with significant increases in middle cerebral artery blood velocity and frontal lobe cerebral oxygenation. Although CBF was not measured in the present study, it could be inferred that the increase in $P_{\mathrm{aC}O_{2}}$ values has influenced our results by attenuating vasopressor‐mediated decreases in $S_{cO_{2}}$. A mean variation in Hb of 1.7 g/L was observed between MAP thresholds, but not between NA and PE. Haemoglobin was identified as a mediator of $S_{cO_{2}}$ in previous studies, but once again, decreases and not increases were associated with cerebral desaturations (Lassnigg et al., 1999).

### 4.4 Clinical significance of data

Interpretations of $S_{cO_{2}}$ in a clinical context must be based on the accepted thresholds for ischaemia, while pondering the possible impacts of known $S_{cO_{2}}$ determinants. In the present study, we consider that variations of parameters other than MAP under CPB were minimal, implying a real and reproducible effect of NA and PE on $S_{cO_{2}}$. Although statistically significant, these values remain clinically insignificant regarding cerebral ischaemia. Cerebral ischaemic thresholds have been defined as a decrease in $S_{cO_{2}}$ of >20% or an absolute $S_{cO_{2}}$ value of <50%, which is far from being the case here, because relative mean $S_{cO_{2}}$ decreases were of 2.0% for both vasopressors (Gaudino et al., 2020). Therefore, such results remain reassuring, because MAP thresholds of 60–80 mmHg are often targeted under CPB in accordance with the 2019 EACTS CPB guidelines (Wahba et al., 2020). Nevertheless, it remains important to consider the mechanism behind vasopressor‐induced decreases in $S_{cO_{2}}$, because a combined effect with other causes of cerebral desaturation could potentially reach a deleterious ischaemic threshold in more vulnerable patients.

### 4.5 Cerebral oxygen saturation and cerebral autoregulation

Interestingly, three patients (11%) in our study showed increases in $S_{cO_{2}}$ values between the 60 and 80 mmHg thresholds. Cerebral autoregulation has not been assessed in the present study, but it remains relevant to consider that its control over CBF presents large interindividual variations (Brassard et al., 2021). It might be possible for these three patients that a MAP of 60 mmHg was located in an accentuated pressure‐passive portion of the cerebral autoregulatory curve that did not allow an optimal coupling between delivery and consumption of oxygen. Hori et al. (2017) have shown that the optimal MAP based on autoregulation indexes was 78 ± 11 mmHg in 614 cardiac surgery patients; however, this value was outside an optimal autoregulatory range in 46% of patients. Such considerations warrant further research, because MAP management based on assessment of cerebral autoregulation might have the potential to reduce neurological dysfunction following cardiac surgery on CPB (Brown et al., 2019; Denault et al., 2018).

### 4.6 Methodological considerations

This study has some limitations that deserve further discussion. No systematic screening of carotid artery stenosis was imposed for recruited patients, potentially leading to confounding factors in the validity of $S_{cO_{2}}$ values. Although the allocation of each vasopressor was randomized, the power of our results remains limited owing to our small sample size. Patients also presented a relatively low burden of comorbidities in the context of cardiac surgery, meaning that the values we obtained might not be reproducible in an already neurologically compromised cohort of patients. Limitations of the cerebral oximetry monitoring, including scalp and skull signal interpretation, might possibly have hindered the validity of our results. However, our data resemble those of the literature and were reproducible among patients in the present study (Holmgaard et al., 2019; Sperna Weiland et al., 2017). Addition of another form of monitoring, such as transcranial Doppler ultrasound, could have allowed us to measure middle cerebral artery blood velocities (a surrogate of CBF), potentially corroborating the vasopressor impact on $S_{cO_{2}}$ and allowing cerebral autoregulation measurements. Quantification of cerebral autoregulation could have also helped in understanding the mechanisms underlying the changes in $S_{cO_{2}}$ reported in the present study. Finally, we conducted this study with meticulous regard for per‐protocol steady‐state management of $S_{cO_{2}}$ determinants, to isolate as much as possible the impact of vasopressors on $S_{cO_{2}}$. Nonetheless, we acknowledge that drawing conclusions from $S_{cO_{2}}$ values can be complex, particularly within a context involving patient comorbidities, effects of anaesthesia and concomitant cardiac surgery.

## 5 CONCLUSION

The $S_{cO_{2}}$ decreased when MAP was elevated from 60 to 80 mmHg solely by means of vasopressor therapy, and the impact on $S_{cO_{2}}$ was comparable for NA and PE during CPB in cardiac surgery. Although statistically significant, this reduction in $S_{cO_{2}}$ was not clinically significant. Mechanisms behind this phenomenon are perplexing, and further research should be directed towards both their understanding and the assessment of optimal MAP management under CPB to minimize postoperative neurological dysfunction.

## AUTHOR CONTRIBUTIONS

Conception or design of the work: Patrice Brassard. Acquisition or analysis or interpretation of data for the work: Edouard Marques, Etienne J. Couture, Jean S. Bussières, Stephan Langevin, Paul Poirier, Pierre Voisine, Manon Caouette and Patrice Brassard. Drafting the work or revising it critically for important intellectual content: Edouard Marques, Etienne J. Couture, Jean S. Bussières, Stephan Langevin, Paul Poirier, Pierre Voisine, Manon Caouette and Patrice Brassard. All authors approved the final version of the manuscript and agree to be accountable for all aspects of the work in ensuring that questions related to the accuracy or integrity of any part of the work are appropriately investigated and resolved. All persons designated as authors qualify for authorship, and all those who qualify for authorship are listed.

## CONFLICT OF INTEREST

Authors report no conflicts of interest.

## Supporting information



Supporting Information

## Data Availability

The data that support the findings of this study are available from the corresponding author upon reasonable request.
