# Supplementary figures and images for: Effects of noradrenaline and phenylephrine on cerebral oxygen saturation during cardiopulmonary bypass in cardiac surgery
Source: Exp Physiol. 2025 Jan 23;110(6):798–808. doi: 10.1113/EP092387 (PMC12128477; doi:10.1113/EP092387)

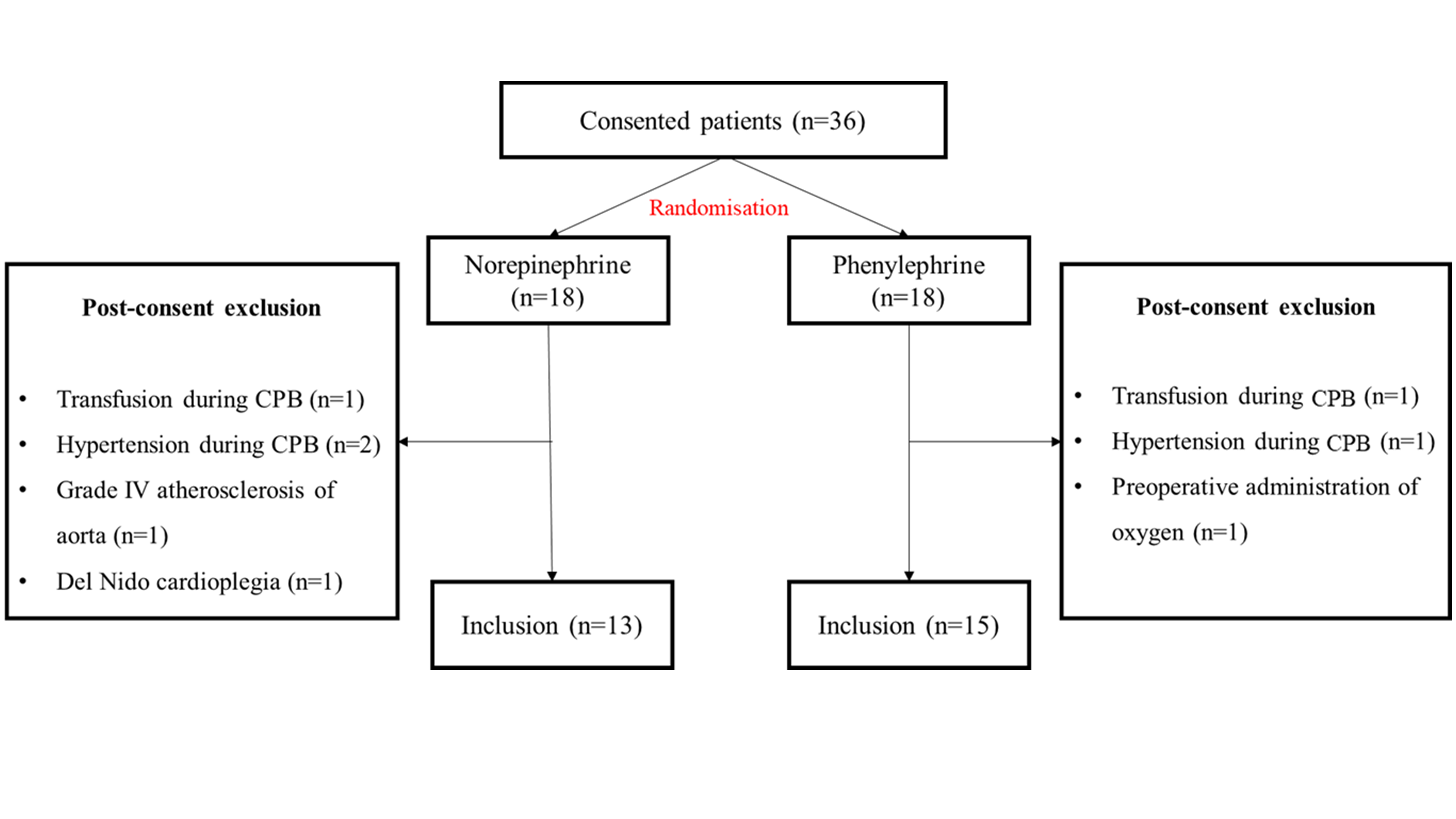

Supplement: Supplementary file 1 — Supporting Information [file EPH-110-798-s001.tif]
